# Dementia-Related Functional Disability in Moderate to Advanced Parkinson’s Disease: Assessment Using the World Health Organization Disability Assessment Schedule 2.0

**DOI:** 10.3390/ijerph16122230

**Published:** 2019-06-24

**Authors:** Jia-Hung Chen, Chien-Tai Hong, Dean Wu, Wen-Chou Chi, Chia-Feng Yen, Hua-Fang Liao, Lung Chan, Tsan-Hon Liou

**Affiliations:** 1 Department of Neurology, Shuang Ho Hospital, Taipei Medical University, New Taipei City 23561, Taiwan; gary.320@hotmail.com (J.-H.C.); chientaihong@gmail.com (C.-T.H.); tingyu02139@gmail.com (D.W.); 2 Department of Neurology, School of Medicine, College of Medicine, Taipei Medical University, Taipei 11031, Taiwan; 3 Taiwan Society of International Classification of Functioning, Disability and Health, TSICF, New Taipei City 23561, Taiwan; y6312002@gmail.com (W.-C.C.); mapleyeng@gmail.com (C.-F.Y.); hfliao@ntu.edu.tw (H.-F.L.); 4 Department of Occupational Therapy, Chung Shan Medical University, Taichung 40201, Taiwan; 5 Department of Public Health, Tzu Chi University, Hualien City 97004, Taiwan; 6 School and Graduate Institute of Physical Therapy, College of Medicine, National Taiwan University, Taipei 10051, Taiwan; 7 Department of Physical Medicine and Rehabilitation, Shuang Ho Hospital, Taipei Medical University, New Taipei City 23561, Taiwan; 8 Department of Physical Medicine and Rehabilitation, School of Medicine, College of Medicine, Taipei Medical University, Taipei 11031, Taiwan; 9 Graduate Institute of Injury Prevention and Control, College of Public Health, Taipei Medical University, Taipei 11031, Taiwan

**Keywords:** Parkinson’s disease (PD), Parkinson’s disease dementia (PDD), World Health Organization Disability Assessment Schedule 2.0 (WHODAS 2.0), International Classification of Functioning, Disability and Health (ICF)

## Abstract

Dementia is a common nonmotor condition among people with moderate or advanced Parkinson’s disease (PD). Undoubtedly, profound motor symptoms cause remarkable impairment in daily activities; however, dementia-related disabilities have not been thoroughly investigated, especially not with consideration of differences according to sex. The present study used the World Health Organization Disability Assessment Schedule 2.0 (WHODAS 2.0) to compare the functional disability between men and women with PD (PwP) with and without dementia. This study employed a registry of disability evaluation and functional assessment using the Taiwan Data Bank of Persons with Disability between July 2012 and October 2018. To investigate dementia-related disability in PwP, 1:1 matching by age and Hoehn-Yahr stage was conducted, which resulted in the inclusion of 1605 study participants in each group. The present study demonstrated that among the six major domains of WHODAS 2.0, the section of “Getting alone with others” was significantly worse in both genders of PwP with dementia; however, a greater disability in fulfilling activities of daily living was only noted in male PwP with dementia but not in their female counterparts. Neither the inability to provide self-care nor participation were significantly different between the sexes. Our findings suggested that deteriorating social relationships were a dementia-related disability in all PwP at the moderate and advanced disease stages. Regarding the performance of activities of daily living, deterioration was related to dementia only in male PwP. Such disabilities could indicate cognitive impairment in people with moderate or advanced PD and could be used as an indicator for the early detection of dementia in PwP by healthcare professionals through the easier functional assessment of the WHODAS 2.0.

## 1. Introduction

Parkinson’s disease (PD) is the second most common neurodegenerative disease and is characterized by the degeneration of dopaminergic neurons in the pars compacta of the substantia nigra. The etiologies of PD include both genetic and environmental factors [1], and its prevalence is higher in Europe (65.6 to 12,500 per 100,000), North America (329.3 per 100,000), and South America (394 per 100,000) than in Asia (51.3 to 176.9 per 100,000) and Africa (20 to 296 per 100,000) [2,3,4,5,6,7]. Currently, more than 4 million people worldwide aged older than 50 years are estimated to be affected, and this figure is expected to double by 2030 [8]. PD is well-known for both its motor and nonmotor symptoms (NMSs) [9]. The motor symptoms are characterized by six core features: tremor, bradykinesia, rigidity, loss of postural reflexes, flexed posture, and freezing, whereas the major NMSs are olfactory dysfunction, cognitive impairment, psychiatric symptoms, sleep disorders, autonomic dysfunction, pain, and fatigue. NMSs have become an increasingly crucial topic because they are more likely to affect the quality of life of people with PD (PwP) [10]. 

Cognitive impairment is not uncommon and poses a great threat to PwP. It may present from the early stage of the disease and progress from mild cognitive impairment (MCI) in PD (PD-MCI) to severe cognitive impairment (PD dementia (PDD)). The most common manifestations of PDD are executive dysfunction, impaired attention, impaired free recall, and visuospatial deficits [11]. The incidence depends on the age, age of PD onset, disease duration, severity of motor symptoms (such as rigidity, gait disturbance, and postural instability), and psychiatric symptoms (such as depression and psychosis) [12]. Approximately 10% of PwP are estimated to transition to PDD annually [13], and a systematic review reported the prevalence of PDD among PwP to be approximately 31.3% [14].

The motor symptoms and NMSs are deleterious to the activities of daily living (ADL) of PwP and thus may accelerate their functional decline through a vicious cycle. For instance, in PwP with dementia, prominent executive dysfunction and psychosis are associated with a greater disability [15]. ADL impairment results in an increase of caregiver burden and economic loss. Caregivers are reported to spend an average of 22 hours per week fulfilling their jobs [16]. In the United States, the economic burden of PD was estimated to exceed US$ 14.4 billion in 2010 (approximately US$ 22,800 per PwP) [17]. Indirect costs, such as productivity loss and the provision of uncompensated care by family members, have been conservatively estimated at US$ 6.3 billion and may account for 45% of total expenses [17,18]. In Japan, direct and indirect costs were also estimated to be US$ 38,000 and US$ 25,000 per PwP [19]. This amount seems to be higher in PwP with dementia because of the greater severity of their disability.

Because PDD is a complicated subtype of PD with a remarkable caregiver and economic burden, early recognition and intervention are urgent and major public health concerns. A conventional PD evaluation called the *Unified Parkinson’s Disease Rating Scale* only barely addresses the cognitive domain and is not familiar to paramedical staff, such as therapists, social workers, and caregivers. The World Health Organization Disability Assessment Schedule 2.0 (WHODAS 2.0) is directly linked with concepts of the International Classification of Functioning, Disability and Health (ICF), and provides a short, simple, and easy method of assessing the standardized disability levels [20]. The WHODAS 2.0 can be applied to all diseases across cultures with a high validity and efficacy. It contains six domains: cognition (understanding and communicating), mobility (moving and getting around), self-care (hygiene, dressing, eating, and staying alone), getting along (interacting with others), life activities (domestic responsibilities, leisure, work, and school), and participation (joining in community activities). In the present study, we aimed to identify the differences in the functional disability between moderate to advanced PwP with and without dementia using the WHODAS 2.0. This could be applied to the early recognition of PDD and the future prevention of functional decline.

## 2. Materials and Methods

### 2.1. Study Design and Participants

In this study, we used the database of the registry of disability evaluation and a functional assessment provided by the Taiwan Data Bank of Persons with Disability, an ICF framework-based database established by the Ministry of Health and Welfare, Taiwan. The registry data from between July 2012 and October 2018 were used. According to the regulations of the Ministry of Health and Welfare, only PwP with modified Hoehn-Yahr stages 3, 4, and 5 are allowed to apply for disability certification to claim corresponding benefits. The ethical approval of this study was approved by the Joint Institutional Review Board of Taipei Medical University (N201805048).

**Figure 1:**
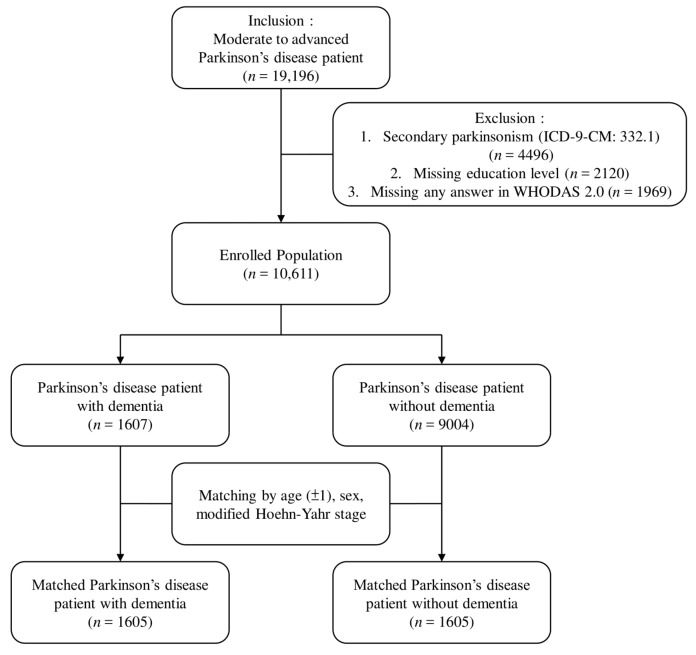
Sample selection flow chart.

As illustrated in Figure 1, the selection process was as follows. In the database, people with the PD diagnosis codes ICD-9-CM 332 or ICD-10-CM G20 were initially included (*n* = 19,196). People were excluded if they had secondary parkinsonism (ICD-9-CM 332.1, *n* = 4496). The remaining 14,700 moderate to advanced PwP merely account for 35% of overall PwP in Taiwan, according to the prevalence survey conducted in 2016 [21]. A previous door-to-door study found that 39% of PwP in Taiwan were at a moderate to advanced stage [22]. Further exclusion of PwP included those who lacked information on the education level, and those who had an incomplete WHODAS 2.0 survey. In total, 10,611 moderate to advanced PwP were enrolled, and those with dementia-related diagnosis codes (ICD-9-CM 290.0, 290.1, 290.2, 290.3, 290.4, 294.1, 331.0, 331.1, 331.2, 331.7, 331.8, and 331.9; and ICD-10-CM F01.50, F01.51, F02.80, F02.81, F03.90, G13.8, G30.0, G30.1, G30.8, G30.9, G31.01, G31.09, G31.1, G31.2, G31.83, G31.85, G31.89, and G31.9) were simultaneously assigned into a PwP with dementia group (*n* = 1607). The remainder were categorized into a PwP without dementia group. Considering the substantial effects of the age, sex, and motor stage of PD on the functional capacity, 1:1 matching based on the age, sex, and modified Hoehn-Yahr stage was conducted to minimize bias. In the end, each group comprised 1605 patients.

### 2.2. Instruments and Measures

The 36-item traditional Chinese version of the WHODAS 2.0 was used, and it was evaluated by several healthcare professionals, including physical therapists, occupational therapists, and social workers in different hospitals in Taiwan. The evaluation period was from July 2012 to October 2018. All healthcare professionals completed the pretest training program, and thus were qualified to conduct the evaluation. Basic demographic data, including the age, sex, residence, work status, education level, family economic status, urbanization level, and modified Hoehn-Yahr stage, were collected before the WHODAS 2.0 assessment. During the WHODAS 2.0 assessment, the functional capacities were evaluated separately in six domains: cognition (domain 1), mobility (domain 2), self-care (domain 3), getting along (domain 4), household activities (domain 5-1), and participation (domain 6). School and work activities (domain 5-2) were not investigated because most moderate to advanced PwP in this study were not enrolled in school or employed. Each domain had between 4 and 8 questions, and each question was scored from 0 to 4 (0 = no difficulty, 1 = mild difficulty, 2 = moderate difficulty, 3 = severe difficulty, and 4 = extreme difficulty or incapability). The total score of all questions in each domain was summed and averaged to produce a final score, which represented the severity in each domain.

### 2.3. Statistical Analysis

Statistical analyses were performed using Statistical Analysis System (version 9.2; SAS Institute Inc., Cary, NC, USA). Chi-square tests were used to analyze the variables of demographic data including the sex, age, education, residence, work status, family economic status, urbanization level, and modified Hoehn-Yahr stage. Wilcoxon rank sum tests were used to compare the differences in the WHODAS 2.0 scores; *p* < 0.05 was considered statistically significant in this study.

## 3. Results

Among all 10,611 PwP, 1607 were comorbid with dementia, and the remaining 9004 were not. The percentage of female PwP was higher in the PwP with dementia group than in the PwP without dementia group (54.45% and 49.46%, respectively; *p* < 0.001). The PwP with dementia group had a significantly higher average age, lower education level, and proportion of participants resident at a care facility. Furthermore, the average Hoehn-Yahr stage was greater in the PwP with dementia group (Table 1).

After the 1:1 matching between PwP with and without dementia, the study participants were again divided into male and female groups (Table 2). For both male and female PwP, no significant difference was observed in the education level between the with and without dementia groups, and higher rates of institutional residence and lower urbanization levels were noted in the dementia group compared with the nondementia group. Regarding the functional capacity of male PwP as assessed by the WHODAS 2.0, dementia was associated with a worse performance in getting along (69.3 ± 28.4 vs. 60.9 ± 30.4, *p* < 0.001) and life activities (76.8 ± 35.6 vs. 70.2 ± 38.7, *p* < 0.001), compared with those without dementia. Female PwP demonstrated a slightly different pattern. Dementia was also associated with a worse performance in getting along with others (69.7 ± 28.3 vs. 62.6 ± 31.8, *p* < 0.001); however, no significant difference was noted in the life activities performance of female PwP with dementia (76.9 ± 35.0 vs. 74.1 ± 37.8, *p* = 0.426) compared to female PwP without dementia. Furthermore, no significant difference existed for either sex in terms of mobility, self-care, or social participation between PwP with and without dementia.

Table 3 presents the scores from each question regarding getting along with others and life activities. Regarding the capacity to get along with others, both male and female PwP with dementia had a greater difficulty in dealing with strangers, maintaining friendships, and getting along with people close to them. Male but not female PwP had a significantly worse ability to make new friends, which was true for those with and without dementia. Moreover, no significant difference existed between the sexes in terms of sexual activity in PwP with or without dementia. The most striking difference between the sexes concerned the life activities performance. Male PwP with dementia had significantly higher scores in all four items compared with their counterparts without dementia. However, no significant difference was observed for any of these questions between female PwP in the with and without dementia groups.

## 4. Discussion

The present study identified the functional differences among moderate to advanced PwP with and without dementia. After matching for age and Hoehn-Yahr stage, dementia in PwP was associated with a greater disability in getting along with people. A greater impairment in the ability to fulfil life activities was only associated with dementia in male PwP. For both sexes, no significant difference existed in mobility, self-care, or social participation between the with and without dementia groups. This revealed that, in certain specific domains, PwP with dementia had a greater ADL impairment compared to those without; furthermore, a difference did exist between the sexes. This knowledge can be applied to a large-scale, community-based survey for the detection of PDD.

Social cognition impairment is an NMS in PwP. It refers to how people process, store, and apply information concerning others and social situations. A study compared PwP with healthy individuals and found that the PwP were impaired in social cognition from the earliest stages of their disease [23]. In the same study, social cognition impairment seemed to be non-significantly associated with other cognitive functions. However, impairment in social relationships is not uncommon in people with dementia. A prospective cohort study in Japan analyzed and followed 13,984 individuals for 10 years and showed that social relationship impairment was highly associated with dementia [24]. In that study, individuals who were married, exchanged support with family members, had contact with friends, participated in community groups, and engaged in paid work had a lower risk of developing dementia. Another systemic review based on 19 studies had similar results [25]. The present study demonstrated that PwP with dementia had a poorer social function than those without dementia, despite PD already being a contributor to social relationship impairment. PwP with dementia encountered more difficulties in making new friends than in getting along with people they were close to. As with other types of dementia, such as Alzheimer’s disease (AD), people with dementia usually lose their ability to learn new skills but preserve long-term memories. This trait seems to also be present in the social function of PwP with dementia. However, our results did not indicate a significant difference in sexual activity between PwP with and without dementia. In a relevant study, sexual disability was present in people with PD, AD, and other types of dementia such as PDD [26]. Considering that PD is already a great contributor to sexual dysfunction, the conversion from PD to PDD may not be able to cause a further significant deterioration in this respect, which is compatible with the findings of our study. Currently, several hypotheses have been proposed for social function in patients with dementia; these include cognitive stimulation [27], easy access to health information and social control for unhealthy behaviors [28], and the reduction of harmful nervous system responses to stress [29]. However, the identification of a definite mechanism requires further study.

Regarding life activities, this study showed that PwP with dementia had a worse performance than PwP without dementia. Rasovska and Rektorova compared instrumental ADL between people with PDD and AD [30] and discovered that motor deficits remained the primary contributor to impairment in PDD. However, the present study matched the severity of PD with modified Hoehn–Yahr stages between the with and without dementia groups, which eliminated the possibility of motor deficits being a confounding factor. Thus, dementia indeed contributed to a greater functional decline in ADL performance for PwP. Stella et al. compared the functional decline in PwP with and without dementia and also found that those with dementia had a greater functional impairment [15]. The functional decline in PD was also shown to be correlated with a cognitive decline similar to AD [31], which explains why PwP with dementia exhibited a greater functional impairment than those without dementia but with the same motor deficits did. However, an unexpected finding was that, in our study, impairment in the performance of life activities did not exhibit significant differences between female PwP with and without dementia. Cognitive decline in female PwP seemed to have less of an effect on instrumental ADL than it did in male PwP. Studies have revealed that male sex was a risk factor for developing PDD with a more severe cognitive decline [32,33], and this more severe cognitive decline might be the cause of a greater instrumental ADL impairment in male PwP with dementia because cognition is a critical contributing factor to functional impairment. However, few studies have focused on the differences in functional decline between the sexes in patients with PDD; furthermore, the definite rationale, such as the cultural background or socioeconomic status, is unclear and requires further study.

Currently, several treatment strategies exist for dementia in PwP, including pharmacological and nonpharmacological strategies. Cholinesterase inhibitors (such as rivastigmine, donepezil, and galantamine) and N–methyl D–Aspartate receptor antagonists (such as memantine) may be considered pharmacological treatments for cognitive decline [34], but their improvements for functional impairment still require further study. Nonpharmacological treatments include physio-exercise therapy, tai chi, qigong, mindfulness meditation, yoga, dance and music therapy, and acupuncture, as well as other modern therapies such as deep brain stimulation, repetitive transcranial magnetic stimulation, near-infrared light therapy, and gene therapy [35]. Some ongoing clinical trials have used psychosocial therapy for PDD [36,37]. These studies have focused on cognitive rehabilitation, but the trials are being processed, and their efficacy must be further evaluated; however, they do provide a silver lining for the treatment of PDD. Nevertheless, the early detection of dementia in people with PD may present an opportunity for redeveloping their functional capacity at early stages by using such treatments.

Our study analyzed numerous PwP with and without dementia, which reliably proved an association between PDD and functional disability. About one third of PwP with and without dementia were included, and the present study could represent most of the PD population in Taiwan. With this evidence, we are able to claim that the WHODAS 2.0 could be an easier and standardized evaluation for clinicians, therapists, social workers, and caregivers to recognize dementia in PwP though functional disability. Education for an early awareness of functional decline in social relationships and life activities for families and caregivers is also a crucial issue. However, this study had some limitations that should be considered. First, it only focused on Taiwanese PwP; other races, cultures, and environments may affect aspects of disability. Second, our database contained no information regarding the treatment strategies, duration, motor subtype, or specific symptoms (i.e., motor fluctuation and complications) of PD, which may have also affected the functional capacity. Third, PwP may have several comorbidities, such as vascular events or psychiatric disorders, that may have affected their daily function performance. Last, diagnoses of PD and dementia may differ among hospitals, which may mislead reviewers to include diseases unrelated to this study, such as dementia with Lewy bodies. However, even with these limitations, our study still demonstrated the functional difference in social relationships and ADL performance between PwP with and without dementia, which could be applied in the early detection of dementia in PwP.

## 5. Conclusions

Functional impairments in social relationships and ADL performance are major indicators of dementia in PwP. This study observed a difference between the sexes in terms of ADL performance but not in terms of social relationships. Because PDD causes a greater functional disability, caregiver burden, and economic loss, the early detection of dementia in PwP is critical. By using the WHODAS 2.0, we were able to evaluate dementia in PwP and conduct early treatment in a quick, simple, and easy manner; accordingly, this method can be adopted to prevent functional decline as well as to reduce the burden on caregivers and economic losses in the future.

## Figures and Tables

**Table 1:** Demographic data of the enrolled population (*n* = 10,611).

Variables	PwPD (*n* = 1607) (*n*/%)	PwPND (*n* = 9004) (*n*/%)	*p*-Value
Sex			<0.001
Male	732/45.55%	4551/50.545%	
Female	875/54.45%	4453/49.46%	
Age			
18–44	5/0.3%	89/1.0%	
45–64	82/5.1%	1926/21.4%	
65 or older	1520/94.6%	6989/77.6%	
Total mean ± SD	77.5 ± 7.2	72.4 ± 9.8	<0.001
Education			<0.001
College or higher	32/2.0%	191/2.1%	
Senior high school	97/6.0%	762/8.5%	
Junior high school	126/7.8%	1380/15.4%	
Primary school	1011/62.9%	5583/62.0%	
No education	341/21.2%	1088/12.1%	
Residence			<0.001
Community dwelling	1315/81.8%	8086/89.8%	
Care facility	292/18.2%	918/10.2%	
Urbanization level			<0.001
Rural	260/16.2%	1037/11.5%	
Suburban	590/36.7%	3141/34.9%	
Urban	757/47.1%	4826/53.6%	
Hoehn-Yahr Stage			<0.001
Stage 3	481/29.9%	3452/38.3%	
Stage 4	693/43.1%	3711/41.2%	
Stage 5	433/26.9%	1841/20.5%	

Abbreviations: PwPD = Parkinson’s disease patient with dementia. PwPND = Parkinson’s disease patient without dementia.

**Table 2:** Demographic data of the matched population (*n* = 3210).

Variables	Male			Female		
	PwPD (*n* = 731) (*n*/%)	PwPND (*n* = 731) (*n*/%)	*p*-Value	PwPD (*n* = 874) (*n*/%)	PwPND (*n* = 874) (*n*/%)	*p*-Value
Age			0.97			1
18–44	3/0.4%	3/0.4%		1/0.1%	1/0.1%	
45–64	52/7.1%	52/7.1%		30/3.4%	31/3.6%	
65 or older	676/92.5%	676/92.5%		843/96.5%	842/96.3%	
Total (mean ± SD)	76.8 ± 7.8	76.8 ± 7.8	0.92	78.2 ± 6.4	78.1 ± 6.4	0.93
Education			0.06			0.17
College or higher	25/3.4%	24/3.3%		7/0.8%	5/0.6%	
Senior high school	70/9.6%	66/9.0%		27/3.1%	34/3.9%	
Junior high school	80/10.9%	70/9.6%		45/5.2%	49/5.6%	
Primary school	482/65.9%	525/71.8%		528/60.4%	563/64.4%	
No education	74/10.1%	46/6.3%		267/30.6%	233/25.5%	
Residence			<0.01			<0.001
Community dwelling	603/82.5%	645/88.2%		711/81.4%	768/87.9%	
Care facility	128/17.5%	86/11.8%		163/18.7%	106/12.1%	
Urbanization level			<0.01			0.01
Rural	104/14.2%	80/10.9%		155/17.7%	123/14.1%	
Suburban	260/35.6%	224/30.6%		330/37.8%	305/34.9%	
Urban	367/50.2%	427/58.4%		389/44.5%	446/51.0%	
Hoehn–Yahr stage			1			1
Stage 3	267/36.5%	267/36.5%		213/24.4%	213/24.4%	
Stage 4	304/41.6%	304/41.6%		389/44.5%	389/44.5%	
Stage 5	160/21.9%	160/21.9%		272/31.1%	272/31.1%	
WHODAS 2.0 score (mean ± SD)						
Cognition (domain 1)	62.7 ± 27.7	50.0 ± 29.1	<0.001	64.5 ± 27.7	54.5 ± 30.1	<0.001
Mobility (domain 2)	59.2 ± 28.3	59.6 ± 27.5	0.88	62.9 ± 28.5	65.2 ± 26.5	0.15
Self-care (domain 3)	40.2 ± 31.6	40.0 ± 30.9	0.90	42.8 ± 33.8	42.1 ± 33.4	0.73
Getting along with people (domain 4)	69.3 ± 28.4	60.9 ± 30.4	<0.001	69.7 ± 28.3	62.6 ± 31.8	<0.001
Life activities (domain 5-1)	76.8 ± 35.6	70.2 ± 38.7	<0.001	76.9 ± 35.0	74.1 ± 37.8	0.43
Participation in society (domain 6)	47.1 ± 24.1	48.7 ± 24.7	0.25	49.0 ± 24.8	50.5 ± 25.2	0.17
Average score	58.0 ± 21.5	53.8 ± 22.4	<0.001	59.9 ± 22.1	57.2 ±22.5	<0.01

Abbreviations: PwPD = Parkinson’s disease patient with dementia. PwPND = Parkinson’s disease patient without dementia.

**Table 3:** Sub-analysis of scores for questions regarding getting along (domain 4) and life activities (domain 5-1).

Questions		Male			Female		
		PwPD (*n* = 731)	PwPND (*n* = 731)	*p*-Value	PwPD (*n* = 874)	PwPND (*n* = 874)	*p*-Value
Getting along (domain 4) (mean ± SD)							
D4.1	Dealing with people you do not know	2.4 ± 1.3	2.0 ± 1.5	<0.001	2.4 ± 1.3	2.1 ± 1.5	<0.001
D4.2	Maintaining friendships	2.6 ± 1.3	2.2 ± 1.4	<0.001	2.6 ± 1.3	2.3 ± 1.5	<0.001
D4.3	Getting along with people who are close to you	1.8 ± 1.3	1.4 ± 1.3	<0.001	1.8 ± 1.4	1.5 ± 1.4	<0.001
D4.4	Making new friends	3.0 ± 1.2	2.7 ± 1.4	<0.001	3.0 ± 1.2	2.9 ± 1.3	0.06
D4.5	Sexual activity	3.1 ± 1.3	3.0 ± 1.4	0.34	3.2 ± 1.2	3.1 ± 1.3	0.56
Life activities (domain 5-1) (mean ± SD)							
D5.1	Taking care of your household responsibilities	2.9 ± 1.5	2.7 ± 1.6	<0.01	2.9 ± 1.4	2.8 ± 1.5	0.16
D5.2	Doing most important household tasks well	2.9 ± 1.5	2.6 ± 1.6	<0.001	3.0 ± 1.4	2.8 ± 1.5	0.11
D5.3	Getting all the household work done that you needed to do	2.9 ± 1.5	2.6 ± 1.6	<0.001	2.9 ± 1.5	2.8 ± 1.5	0.34
D5.4	Getting your household work done as quickly as needed	3.0 ± 1.4	2.7 ± 1.6	<0.001	3.0 ± 1.4	2.9 ± 1.5	0.06

Abbreviations: PwPD = Parkinson’s disease patient with dementia. PwPND = Parkinson’s disease patient without dementia.
